# Primary pulmonary NUT carcinoma with NSD3–NUTM1 fusion treated with dose-escalated adaptive radiotherapy and multimodal therapy: case report

**DOI:** 10.3389/fonc.2026.1827995

**Published:** 2026-05-20

**Authors:** Cenk Umay, Mehmet Çağrı Duymaz, Zeynep Bayramoğlu, Volkan Semiz, Umut Basan, Kübra Canaslan, Tuğba Yavuzşen, Emrullah Rıza Çetingöz, Ayşe Nur Demiral

**Affiliations:** 1 Department of Radiation Oncology, Dokuz Eylül University Faculty of Medicine, Izmir, Türkiye; 2 Department of Pathology, Dokuz Eylül University Faculty of Medicine, Izmir, Türkiye; 3 Clinic of Radiation Oncology, İzmir City Hospital, Izmir, Türkiye; 4 Department of Medical Oncology, Mehmet Akif İnan Training and Research Hospital, Şanlıurfa, Türkiye; 5 Department of Medical Oncology, Dokuz Eylül University Faculty of Medicine, Izmir, Türkiye

**Keywords:** adaptive radiotherapy, case report, NSD3–NUTM1 fusion, NUT carcinoma, primary pulmonary neoplasm

## Abstract

Primary pulmonary NUT carcinoma (PPNC) is an exceptionally rare and aggressive malignancy driven by NUTM1 rearrangements, with a median overall survival (OS) of 5–9 months. While BRD4–NUTM1 accounts for approximately 70% of cases, the rarer NSD3–NUTM1 fusion, reported in approximately 6% of NUT carcinomas, is poorly characterized, and data on optimal radiotherapy (RT) techniques and adaptive strategies remain extremely limited. We describe a 27-year-old man with PPNC harboring an NSD3–NUTM1 fusion (cT4N2M1c), confirmed by next-generation sequencing (NGS) following equivocal NUT immunohistochemistry. Given rapidly progressive post-obstructive atelectasis and airway compromise, a staged, adaptive thoracic RT strategy was employed: urgent hypofractionated IMRT was initiated with 5 × 3 Gy, followed by 15 × 2 Gy using the initial plan, as early cone-beam CT showed insufficient anatomical change for immediate replanning. Subsequent tumor regression and right lung re-expansion enabled repeat CT simulation and adaptive volumetric modulated arc therapy (VMAT) for the final 9 × 2 Gy, achieving a cumulative tumor-effect EQD2α/β10Gy of 64.25 Gy. Interim PET/CT revealed in-field thoracic regression but widespread systemic progression; cisplatin–etoposide was added during the remaining RT course, followed by multisite palliative RT and consolidation cisplatin–etoposide–ifosfamide chemotherapy. Despite an initial metabolic response, hepatic progression precluded planned immunotherapy. OS was 8.8 months. This case suggests that staged, dose-escalated adaptive thoracic RT is feasible in selected patients with disseminated PPNC and compromised pulmonary function, providing durable in-field control and meaningful symptom palliation as part of a multimodal treatment approach. Molecular confirmation by NGS is essential for non-BRD4 fusions, and systemic therapy should be incorporated as early as safely feasible to address the high risk of distant dissemination.

## 1 Introduction

NUT carcinoma is a rare, highly aggressive squamous malignancy driven by chromosomal rearrangements involving the NUTM1 gene, most commonly the t(15;19) translocation (1, 2). The resultant fusion proteins, most frequently BRD4–NUT, interact with p300, creating hyperacetylated chromatin megadomains that block terminal differentiation and sustain proliferation (3). Primary pulmonary NUT carcinoma (PPNC) has been reported in fewer than 100 cases (4). Although BRD4–NUTM1 accounts for approximately 70% of cases, our patient harbored the rarer NSD3–NUTM1 fusion (~6% of cases) (5, 6). Despite multimodal therapy, median OS is generally 5–9 months, and the 5-year OS has been reported as 7.1% (7, 8). Optimal management strategies, particularly the role of RT, remain undefined.

Here, we describe a case of PPNC harboring the rare NSD3–NUTM1 fusion, emphasizing the adaptive RT strategy, rationale for dose escalation, and treatment response. To our knowledge, this is the first reported case of an adult patient with PPNC from Türkiye, and this report provides one of the most detailed accounts to date of RT management in this rare entity.

## 2 Case description

### 2.1 Initial presentation and imaging

**Figure 1:**
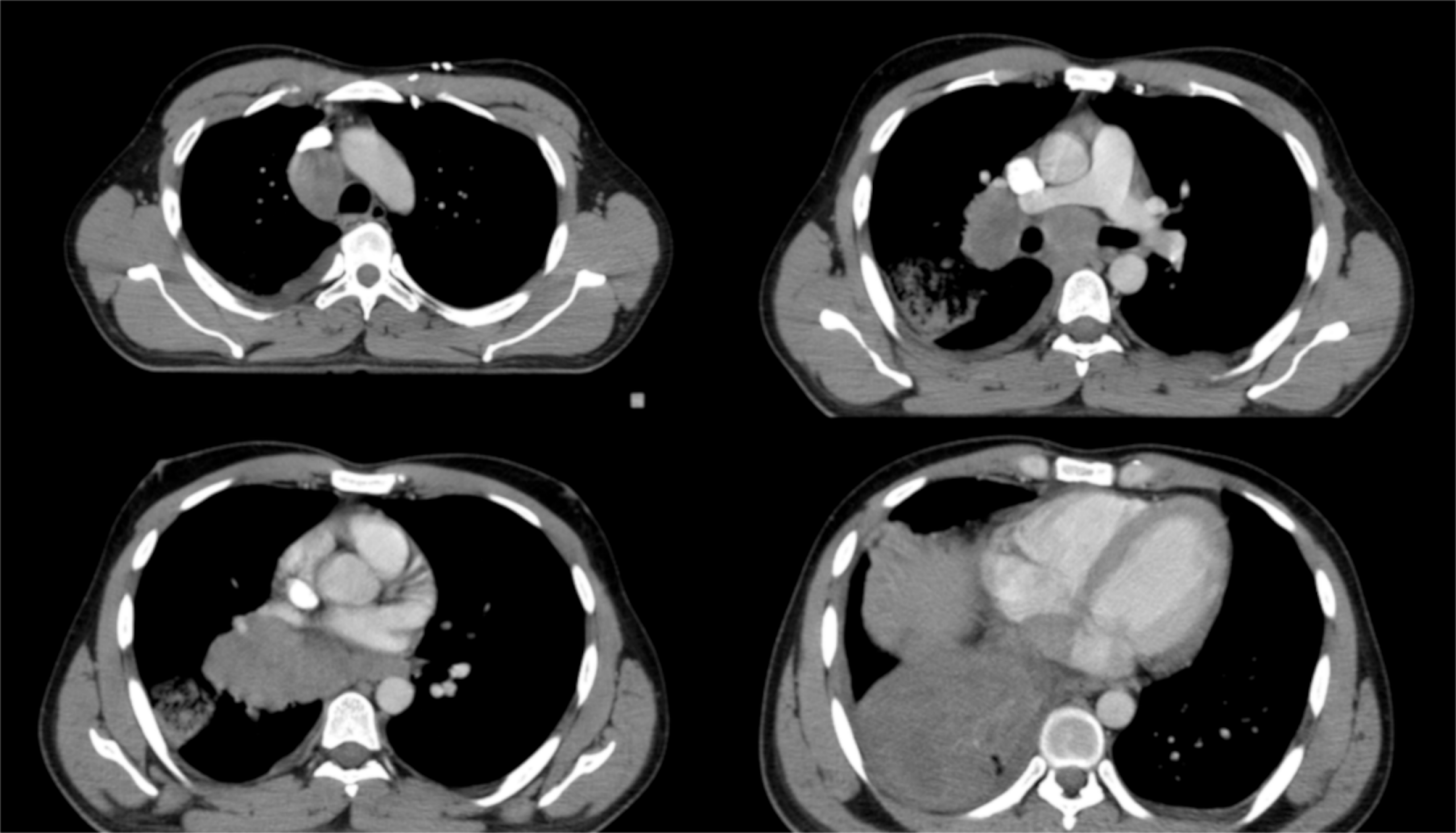
Axial CT-simulation images at diagnosis showing a centrally located right-sided mass invading the mediastinum and right pulmonary vasculature.

A 27-year-old man with a 10-pack-year smoking history (quit after diagnosis) and unremarkable medical and family histories presented in January 2023 with cough and intermittent hemoptysis that had persisted for 3 months. He had no relevant past medical history, no prior thoracic radiotherapy or systemic anticancer treatment, and no known family history of malignancy or hereditary cancer syndrome. Contrast-enhanced thoracic CT revealed a 10-cm central right lung mass invading adjacent mediastinal structures, obliterating the right main bronchus, and causing extensive atelectasis of the right lower lobe. Necrotic ipsilateral and contralateral mediastinal and right hilar lymph nodes (up to 3.5 cm), pleural irregularities, and right main pulmonary artery invasion with subsegmental emboli were present (**Figure 1**).

### 2.2 Pathologic and molecular diagnosis

**Figure 2:**
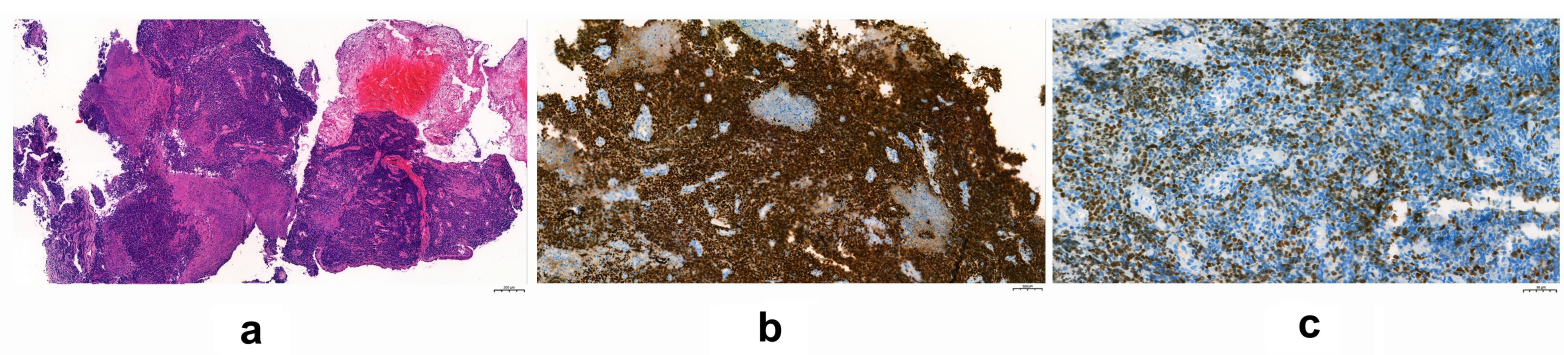
Histopathologic and immunohistochemical features: **(a)** Poorly differentiated monotonous tumor cells with abrupt keratinization (H&E); **(b)** Diffuse nuclear p63 expression; **(c)** Weak nuclear NUT expression confirming diagnosis.

Bronchoscopy revealed near-total obliteration of the right middle lobe bronchus. Histopathological examination of endobronchial biopsies and EBUS-TBNA samples from stations 4R and 7 identified a poorly differentiated carcinoma composed of atypical cell sheets with prominent nucleoli, extensive necrosis, and a neutrophil-rich inflammatory background (**Figure 2a**). A distinctive feature was the presence of abrupt keratinization. Immunohistochemical (IHC) analysis showed diffuse positivity for pancytokeratin (AE1/AE3), p40, and p63, supporting squamous differentiation (**Figure 2b**), while TTF-1 staining was focal and weak. Neuroendocrine markers (synaptophysin, chromogranin, and CD56) were negative, and SMARCA4 (BRG1) expression was preserved. IHC for NUT (clone C52B1) demonstrated weak, focal nuclear staining in approximately 10–15% of tumor cells, interpreted as an equivocal result (**Figure 2c**).

In the differential diagnosis, poorly differentiated squamous cell carcinoma was initially considered; however, the abrupt keratinization pattern and NUT immunoreactivity were atypical for a conventional SCC. Small cell carcinoma was excluded based on the absence of nuclear molding and fine chromatin, as well as negative neuroendocrine staining. Despite morphological overlap, basaloid carcinoma was ruled out based on NUT expression. SMARCA4-deficient thoracic neoplasms were also excluded by the lack of rhabdoid morphology and the maintenance of SMARCA4 expression. Furthermore, the absence of glandular differentiation and strong squamous marker expression rendered adenocarcinoma unlikely despite focal TTF-1 positivity. To reach a definitive diagnosis, Next-Generation Sequencing (NGS) was utilized. A targeted RNA-based fusion panel identified an NSD3–NUTM1 fusion involving NSD3 exon 7 and NUTM1 exon 3, confirming the diagnosis of NUT carcinoma. This case exemplifies that molecular confirmation may be necessary even when morphology strongly suggests NUT carcinoma, particularly in tumors harboring non-BRD4 fusion partners, which may exhibit weaker immunohistochemical staining (9).

### 2.3 Staging and work-up

PET/CT (January 2023) demonstrated intense FDG uptake in the primary lesion and mediastinal/right hilar nodes (SUVmax up to 23.2), a cortical abnormality in the left sixth rib (SUVmax 5.6), and a hypermetabolic focus in the left ischium (SUVmax 9.8), suspicious for early osseous metastasis. Brain MRI was normal. Staging was cT4N2M1c.

### 2.4 Radiotherapy strategy and adaptive planning

**Figure 3:**
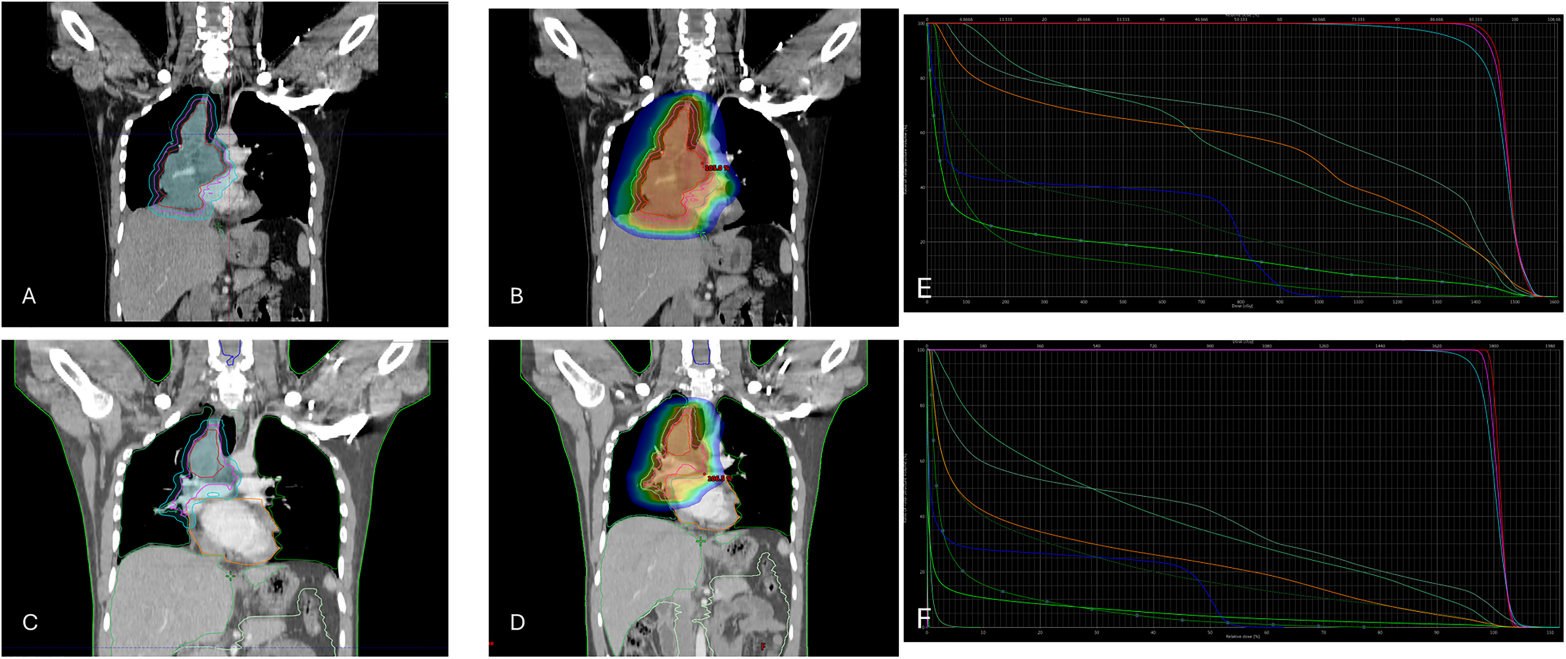
Dose distributions and dose volume histograms of the staged adaptive thoracic radiotherapy strategy. **(A)** Initial planning CT with target and selected organ-at-risk contours. **(B)** Initial IMRT dose distribution. **(C)** Repeat planning CT after tumor regression and right lung re-expansion with adaptive target and organ-at-risk contours. **(D)** Adaptive VMAT dose distribution. **(E)** Dose volume histogram of the initial plan. **(F)** Dose–volume histogram of the adaptive VMAT plan.

In February 2023, the multidisciplinary tumor board reviewed the case. Performance status was good (Karnofsky 90%), but pulmonary function tests showed moderate impairment (FEV1 61%, DLCO 49%). Given rapidly evolving post-obstructive atelectasis, urgent RT was initiated. CT-simulation was performed with a wing board, 3-mm slice thickness, and free-breathing. The initial thoracic plan was delivered with 5-field IMRT, and the adaptive plan with VMAT; both used 15-MV photons (**Figure 3**). The RT strategy was staged and adaptive:

Target volume definition: The gross tumor volume (GTV) was delineated on the planning CT with reference to co-registered diagnostic contrast-enhanced CT and ¹^8^F-FDG PET/CT. The clinical target volume (CTV) was generated by adding a 5-mm margin to the GTV and including relevant nodal regions, namely stations 4R, 5, 7, 10R, and 11R, and was manually edited to conform to anatomical boundaries and expected patterns of local spread. The planning target volume (PTV) was created using an additional isotropic 5-mm setup margin. Daily cone-beam CT image guidance was performed throughout treatment.

Phase 1 (Initial decompressive RT): An urgent hypofractionated course of 15 Gy in 5 fractions was initiated to address airway compromise. Concurrent chemoradiotherapy (CRT) was deferred at this stage because of the extensive thoracic target volume, impaired baseline pulmonary function, and unfavorable projected OAR doses for definitive-dose CRT. After the initial 5 fractions, early CBCT did not demonstrate sufficient tumor regression or right lung re-expansion to justify immediate replanning; therefore, thoracic RT was continued with conventional fractionation using the initial plan for an additional 30 Gy in 15 fractions.

Phase 2 (Adaptive dose escalation): Subsequent CBCT demonstrated tumor regression and right lung re-expansion, prompting repeat CT simulation and adaptive replanning. The adaptive plan was delivered with VMAT for the final 18 Gy in 9 fractions, enabling locally dose-escalated thoracic radiotherapy aimed at durable local control in the setting of M1c disease. The cumulative thoracic physical dose was 63 Gy, delivered as 5 × 3 Gy followed by 15 × 2 Gy and adaptive VMAT 9 × 2 Gy. Cumulative thoracic dose was calculated by physical summation of the delivered phases. EQD2 was estimated using the linear-quadratic model with α/β = 10 Gy for tumor effect and α/β = 3 Gy for late-responding tissues, yielding cumulative EQD2 values of 64.25 Gy and 66 Gy, respectively. Adaptive replanning resulted in substantial target-volume reductions, with GTV decreasing from 365 cm³ to 45 cm³, CTV from 568 cm³ to 160 cm³, and PTV from 880 cm³ to 312 cm³. In normalized 60 Gy/30-fraction plan comparisons, adaptive VMAT reduced mean lung dose, lung V20, spinal cord Dmax, heart mean dose, and esophageal mean dose compared with the initial plan (**Table 1**).

**Table 1:** Target-volume changes and normalized OAR dosimetric comparison between initial and adaptive thoracic RT plans.

Parameter	Phase 1	Phase 2	Reduction
*Target volumes*			
GTV volume, cm³	365	45	87.7%
CTV volume, cm³	568	160	71.8%
PTV volume, cm³	880	312	64.5%
*Target coverage, actual delivered plans*			
CTV D_95_	94.7%	98.4%	—
PTV D_95_	89.0%	96.0%	—
*Organ-at-risk parameters, normalized to 60 Gy in 30 fractions*			
Mean lung dose, Gy	16.5	12.3	25.5%
Lung V_20_, %	34	23	32.4%
Spinal cord D_max, Gy	42	37	11.9%
Heart mean dose, Gy	32	14.2	55.6%
Esophagus D_max, Gy	63.7	64.0	No reduction
Esophagus mean dose, Gy	38.5	23.7	38.4%

Target-volume reductions were calculated between the initial and adaptive planning CTs. OAR dosimetric comparisons were performed after normalizing both the initial and adaptive plans to 60 Gy in 30 fractions, in order to illustrate the dosimetric effect of adaptive replanning independent of the actually delivered fractionation. Reduction was calculated as: (Phase 1 value − Phase 2 value)/Phase 1 value × 100. GTV, gross tumor volume; CTV, clinical target volume; PTV, planning target volume; OAR, organ at risk; VMAT, volumetric modulated arc therapy.

### 2.5 Treatment course, response, and outcome

Concurrent systemic therapy was deferred during Phase 1 because of the extensive pre-decompression target volume (PTV_1_ 880 cm³), near-tolerance lung and esophageal doses, and the patient’s impaired pulmonary reserve (FEV_1_ 61%, DLCO 49%), which precluded safe administration of cytotoxic chemotherapy. During the second week of Phase 1, severe lumbar pain prompted spinal imaging that disclosed new lytic lesions at T10–L2. After fraction 14, an interim ¹^8^F-FDG PET/CT (March 2023) demonstrated metabolic regression of the in-field thoracic disease but widespread systemic progression: hepatic, adrenal, and renal metastases; para-pancreatic lymphadenopathy; extensive new osseous metastases with pathological fracture; and bilateral pleural effusions.

These findings prompted multidisciplinary escalation. Phase 1 was completed as planned. Adaptive replanning on a new planning CT then documented marked tumor regression and right lung re-expansion (88% GTV reduction, 65% PTV reduction). The reduced target volume and improved respiratory mechanics permitted safe systemic intensification, and concurrent cisplatin 50 mg/m² (days 1, 8) plus etoposide 50 mg/m² (days 1–5) was introduced at fraction 1 of Phase 2 VMAT, completed across fractions 1–9 without dose-limiting toxicity or unplanned treatment interruption. Palliative RT to the T10–L2 vertebrae (10 × 3 Gy = 30 Gy) was delivered concurrently to address the new lytic spinal disease.

After completion of thoracic chemoradiotherapy, systemic therapy was intensified to consolidation cisplatin–etoposide–ifosfamide (ICE; cisplatin 20 mg/m² days 1–5, etoposide 100 mg/m² days 1–5, ifosfamide 1000 mg/m² days 1–2 with mesna 1000 mg/m² days 1–2 for uroprotection). Palliative RT to L3 and the bilateral sacroiliac joints (6 × 4 Gy = 24 Gy) was delivered for pain control during the first consolidation cycle. The complete radiotherapy summary is provided in **Table 2**.

**Table 2:** Summary of radiotherapy delivered.

Target	Dose/fractions	Intent/technique	Notes
Thoracic (Phase 1)	15 Gy/5 fx + 30 Gy/15 fx	Definitive/IMRT	Decompression; airway compromise
Thoracic (Phase 2)	18 Gy/9 fx	Definitive/VMAT	Adaptive replanning after re-expansion; concurrent CRT
Thoracic Total	63 Gy (EQD2*α/β*3Gy: 66 Gy; EQD2*α/β*10Gy: 64.25 Gy)	—	Locally dose-escalated thoracic RT for definitive local control
T10–L2 vertebrae	30 Gy/10 fx	Palliative/Hypofractionated/IMRT	Concurrent with thoracic CRT, pain relief
L3–SI joints	24 Gy/6 fx	Palliative/Hypofractionated/IMRT	During consolidation CT, pain relief
Left 6th rib + left femur	Incomplete	Palliative/IMRT and 3D-CRT	Discontinued due to clinical deterioration

CRT, chemoradiotherapy; CT, chemotherapy; EQD2, equivalent dose in 2 Gy fractions; fx, fractions; IMRT, intensity-modulated radiation therapy; SI, sacroiliac.

Bold values indicate the cumulative total radiotherapy dose delivered to the thoracic target volume.

No CTCAE grade ≥3 acute RT-related toxicity was observed during thoracic RT, and no unplanned RT interruption occurred. The patient developed grade 1–2 radiation esophagitis/odynophagia and grade 1 fatigue, both of which were managed conservatively. No supplemental oxygen was required at any time during the thoracic RT course. Baseline performance status was preserved, with Karnofsky Performance Status remaining 90 throughout treatment. Lumbar and metastatic bone pain were initially treated with non-steroidal anti-inflammatory drugs; transdermal fentanyl 50 μg/h was subsequently added, leading to marked improvement in overall pain, including odynophagia-related discomfort. Zoledronic acid was also administered for symptomatic osseous metastatic disease. Overall, the patient experienced clinically meaningful relief of dyspnea during thoracic RT and substantial palliation of bone pain after spinal RT.

In June 2023, PET/CT showed a marked metabolic response with a single newly detected hepatic lesion consistent with oligoprogression. Five total cycles of cisplatin–etoposide–ifosfamide were administered. August 2023 PET/CT demonstrated hepatic progression. PD-L1 expression was 3%; pembrolizumab was planned but could not be initiated due to evolving hepatic failure. Epidural intracranial metastases developed subsequently. In the final weeks of life, a new left femoral metastasis was identified, and the previously untreated left sixth rib lesion had become symptomatic; palliative RT was initiated to both sites concurrently using IMRT and 3D-CRT, but the course was discontinued before completion owing to rapid clinical deterioration. The patient died 8.8 months after diagnosis. The clinical timeline is summarized in **Table 3**.

**Table 3:** Clinical timeline.

Date	Category	Clinical event
Oct 2022	*Symptoms*	Onset of cough and intermittent hemoptysis (3-month history).
Jan 2023	*Imaging*	Contrast-enhanced thoracic CT: 10-cm central right lung mass with right main bronchus obstruction and right lower lobe atelectasis.
Jan 2023	*Diagnostic*	Bronchoscopy and EBUS-TBNA (stations 4R, 7); biopsies of primary mass and mediastinal nodes.
Jan 2023	*Pathology*	Poorly differentiated carcinoma; equivocal NUT immunohistochemistry; full IHC panel performed.
Jan 2023	*Molecular*	Next-generation sequencing confirmed NSD3 (exon 7) – NUTM1 (exon 3) fusion.
Jan 2023	*Staging*	FDG-PET/CT and brain MRI; final stage cT4N2M1c (osseous metastases at left 6th rib and left ischium).
Feb 2023	*Tumor board*	Multidisciplinary tumor board; staged adaptive RT strategy approved. KPS 90%; FEV_1_ 61%; DLCO 49%.
Feb 2023	*RT — Phase 1*	Urgent decompressive thoracic IMRT, 15 Gy/5 + 30 Gy/15 fractions; concurrent chemotherapy deferred.
Mar 2023	*Adaptive replan*	Cone-beam CT confirmed right lung re-expansion; new planning CT acquired and adaptive replanning performed.
Mar 2023	*RT — Phase 2*	Definitive dose-escalated thoracic VMAT, 18 Gy/9 fractions (2 Gy/fx). Cumulative EQD2: 66 Gy (α/β = 3); 64.25 Gy (α/β = 10).
Mar 2023	*Clinical event*	New-onset severe lumbar pain; spinal MRI revealed lytic lesions at T10–L2.
Mar 2023	*Imaging*	Interim PET/CT after fraction 14: in-field thoracic regression; widespread systemic progression (hepatic, adrenal, renal, peritoneal, osseous).
Mar 2023	*Systemic Tx*	Concurrent CRT with cisplatin 50 mg/m² (D1, D8) + etoposide 50 mg/m² (D1–5) initiated during Phase 2.
Mar 2023	*Palliative RT*	Spinal RT to T10–L2 vertebrae, 30 Gy/10 fractions (3 Gy/fx).
Apr–Jun 2023	*Systemic Tx*	Consolidation chemotherapy: cisplatin–etoposide–ifosfamide (PEI) × 5 cycles.
Apr 2023	*Palliative RT*	Spinal RT to L3 and bilateral sacroiliac joints, 24 Gy/6 fractions (4 Gy/fx).
Jun 2023	*Imaging*	PET/CT: marked metabolic response with a single new hepatic lesion (oligoprogression).
Aug 2023	*Progression*	PET/CT: hepatic progression. PD-L1 expression 3%; pembrolizumab planned.
Aug 2023	*Systemic Tx*	Pembrolizumab could not be initiated due to evolving hepatic failure.
Aug 2023	*Progression*	Development of epidural intracranial metastases.
Sep–Oct 2023	*Palliative RT*	Attempted palliative RT to left sixth rib and left femur (32,5 Gy/13 fractions); discontinued before completion due to clinical deterioration
Oct 2023	*Outcome*	Death at 8.8 months from initial diagnosis.

CRT, chemoradiotherapy; CT, computed tomography; EBUS-TBNA, endobronchial ultrasound–guided transbronchial needle aspiration; EQD2, equivalent dose in 2 Gy fractions; FDG, fluorodeoxyglucose; FEV_1_, forced expiratory volume in 1 second; DLCO, diffusing capacity of the lungs for carbon monoxide; IHC, immunohistochemistry; IMRT, intensity-modulated radiation therapy; KPS, Karnofsky performance status; MRI, magnetic resonance imaging; PD-L1, programmed death–ligand 1; PEI, cisplatin–etoposide–ifosfamide; PET, positron emission tomography; RT, radiotherapy.

## 3 Discussion

This case details a multifaceted management strategy for PPNC harboring the rare NSD3–NUTM1 fusion (~6% of cases) (5, 6). The observed OS of 8.8 months was clinically meaningful compared with the registry median of 4.4 months for thoracic primaries (Group C), but should be interpreted cautiously given the single-case design and multimodal treatment course (5). Our experience highlights two critical pillars: the need for molecular confirmation via NGS when immunohistochemistry is equivocal, and the clinical potential of staged, dose-escalated adaptive RT to achieve local control and palliation in this rapidly progressive entity.

The initial urgent hypofractionated phase of 5 × 3 Gy addressed the immediate need for airway decompression. Because early CBCT did not show sufficient anatomical changes to warrant immediate replanning, thoracic RT was continued with conventional fractionation using the initial plan for an additional 15 × 2 Gy. Subsequent tumor regression and right lung re-expansion enabled repeat CT simulation and adaptive VMAT replanning for the final 9 × 2 Gy, allowing locally dose-escalated thoracic radiotherapy aimed at durable local control in the setting of M1c disease. The cumulative tumor-effect EQD2α/β10Gy was 64.25 Gy, approaching the lower range of the recommended definitive dose of 65–70 Gy (10, 11), while the EQD2α/β3Gy for late-responding tissues was 66 Gy. In the largest PPNC series, CRT yielded a mean OS of 13.2 months compared with 5.6 months for chemotherapy alone, with over half of CRT-treated patients surviving beyond 1 year (4). These data support integrating RT into multimodal management, particularly for local control and symptom palliation, although survival outcomes cannot be attributed solely to RT.

This case also highlights the importance of early concurrent chemotherapy. Although CRT was initially deferred due to lung tolerance constraints, interim PET/CT revealed in-field regression but widespread distant progression—a critical lesson: concurrent chemotherapy should be incorporated as early as safely feasible, even in patients with borderline pulmonary reserve, to address the profound propensity for systemic dissemination (7, 12).

The rapid hepatic progression despite an initial metabolic response and the inability to initiate pembrolizumab due to evolving hepatic failure illustrate the narrow therapeutic window in NUT carcinoma. The low PD-L1 expression (3%) is consistent with its typically cold tumor microenvironment (13), though isolated durable responses to immune checkpoint inhibitors have been reported (14, 15).

The main strengths of this report are the molecular confirmation of a rare NSD3–NUTM1 fusion, the detailed description of a staged adaptive thoracic RT strategy, the inclusion of target-volume changes and dosimetric comparisons, and the reporting of symptom response and treatment tolerability. These details may be useful for radiation oncologists facing similar cases with central airway compromise and limited pulmonary reserve. However, several limitations should be acknowledged. First, this is a single-case report, and the findings cannot be generalized or used to establish a standard treatment approach. Second, the patient received multimodal therapy, including thoracic RT, concurrent cisplatin–etoposide, multisite palliative RT, and consolidation chemotherapy; therefore, the patient’s survival outcomes cannot be attributed solely to adaptive RT. Third, the short follow-up, the absence of autopsy confirmation, and limited access to raw NGS sequencing files limit the ability to fully characterize disease biology and patterns of failure. Therefore, the principal demonstrated benefit of the staged adaptive RT approach in this case should be interpreted as improved local control, airway decompression, relief of dyspnea, and symptom palliation rather than proven survival prolongation.

## 4 Patient perspective

The patient had deceased before manuscript preparation. Written informed consent for participation and publication was obtained from the patient’s family (legal representatives). The family reported significant subjective improvement in breathing capacity and bone pain control during the radiotherapy course.

## Data Availability

The data supporting the findings of this case report are included within the article. A third-party laboratory performed the NGS analysis, and the authors do not have access to the raw sequencing files. Requests to access these datasets should be directed to mehmetcagri.duymaz@deu.edu.tr.

## References

[1] French C . Nut midline carcinoma. Nat Rev Cancer. (2014) 14:149–50. doi: 10.1038/nrc3659. PMID: 25688404

[2] French CA Miyoshi I Kubonishi I Grier HE Perez-Atayde AR Fletcher JA . Brd4-nut fusion oncogene: a novel mechanism in aggressive carcinoma. Cancer Res. (2003) 63:304–7. 12543779

[3] Reynoord N Schwartz BE Delvecchio M Sadoul K Meyers D Mukherjee C . Oncogenesis by sequestration of CBP/p300 in transcriptionally inactive hyperacetylated chromatin domains. EMBO J. (2010) 29:2943–52. doi: 10.1038/emboj.2010.176. PMID: 20676058 PMC2944051

[4] Yuan J Xu Z Guo Y . Diagnosis, treatment, and prognosis of primary pulmonary nut carcinoma: a literature review. Curr Oncol. (2022) 29:6807–15. doi: 10.3390/curroncol29100536. PMID: 36290813 PMC9600367

[5] Chau NG Ma C Danga K French CA Bradner JE Drappatz J . An anatomical site and genetic-based prognostic model for patients with nuclear protein in testis (NUT) midline carcinoma: analysis of 124 patients. Jnci Cancer Spectr. (2019) 4:pkz094. doi: 10.1093/jncics/pkz094. PMID: 32328562 PMC7165803

[6] Mao N Liao Z Wu J Liang K Wang S Qin S . Diagnosis of nut carcinoma of lung origin by next-generation sequencing: case report and review of the literature. Cancer Biol Ther. (2019) 20:150–6. doi: 10.1080/15384047.2018.1523852. PMID: 30307375 PMC6343686

[7] Giridhar P Mallick S Kashyap L Rath GK . Patterns of care and impact of prognostic factors in the outcome of nut midline carcinoma: a systematic review and individual patient data analysis of 119 cases. Eur Arch Otorhinolaryngol. (2018) 275:815–21. doi: 10.1007/s00405-018-4882-y. PMID: 29356890

[8] Bauer DE Mitchell CM Strait KM Lathan CS Stelow EB Luer SC . Clinicopathologic features and long-term outcomes of nut midline carcinoma. Clin Cancer Res. (2012) 18:5773–9. doi: 10.1158/1078-0432.CCR-12-1153. PMID: 22896655 PMC3473162

[9] Zhang Y Zhang Q Hao Y Chen X Liu Y Wang Z . International guidelines on the diagnosis and treatment of nut carcinoma. Innovation. (2026) 7:101068. doi: 10.1016/j.xinn.2025.101068. PMID: 41737331 PMC12925906

[10] Lemelle L Flaadt T Fresneau B Bielle F Carton M Velasco V . Nut carcinoma in children and adolescents: expert European standard clinical practice harmonized recommendations. J Pediatr Hematol Oncol. (2023) 45:165–73. doi: 10.1097/MPH.0000000000002568. PMID: 36219702

[11] Luo J Bishop JA DuBois SG French CA Bhatt RS Christensen JG . Hiding in plain sight: nut carcinoma is an unrecognized subtype of squamous cell carcinoma of the lungs and head and neck. Nat Rev Clin Oncol. (2025) 22:292–306. doi: 10.1038/s41571-025-00986-3. PMID: 39900969 PMC12077380

[12] Luo J Sanchez M Lee E Schenk EL Bauer TM Patel MR . Initial chemotherapy for locally advanced and metastatic nut carcinoma. J Thorac Oncol. (2024) 19:829–38. doi: 10.1016/j.jtho.2023.12.022. PMID: 38154515 PMC11081848

[13] Kroening G Luo J Evans MG Forrest SJ Davis EM Pinto N . Multiomic characterization and molecular profiling of nuclear protein in testis carcinoma. Jco Precis Oncol. (2024) 8:e2400334. doi: 10.1200/PO.24.00334. PMID: 39447095 PMC11520346

[14] Ng JKW Wong ECY So TCY Wong RTS . Case report: long term remission of metastatic sinonasal nut carcinoma after palliative radiotherapy and immunotherapy in an elderly patient. Front Oncol. (2025) 14:1412070. doi: 10.3389/fonc.2024.1412070. PMID: 39839761 PMC11746124

[15] Haebe S Schuebbe G Jurmeister P French CA Blay J-Y Le Tourneau C . Adding checkpoint inhibitors to first-line chemotherapy for nut carcinoma patients. NPJ Precis Oncol. (2025) 9:26. doi: 10.1038/s41698-024-00768-7. PMID: 39863772 PMC11762287

